# Pyrosequencing-based comparative genome analysis of the nosocomial pathogen *Enterococcus faecium *and identification of a large transferable pathogenicity island

**DOI:** 10.1186/1471-2164-11-239

**Published:** 2010-04-14

**Authors:** Willem van Schaik, Janetta Top, David R Riley, Jos Boekhorst, Joyce EP Vrijenhoek, Claudia ME Schapendonk, Antoni PA Hendrickx, Isaäc J Nijman, Marc JM Bonten, Hervé Tettelin, Rob JL Willems

**Affiliations:** 1 Department of Medical Microbiology, University Medical Center Utrecht, Heidelberglaan 100, 3584 CX Utrecht, The Netherlands; 2 Institute for Genome Sciences, Department of Microbiology and Immunology, University of Maryland School of Medicine, BioPark II Room 629, 801 West Baltimore Street, Baltimore, MD 21201, USA; 3 Department of Biology, Faculty of Science, Utrecht University, Padualaan 8, 3584 CH Utrecht, The Netherlands; 4 Hubrecht Institute for Developmental Biology and Stem Cell Research, Cancer Genomics Center, Uppsalalaan 8, 3584 CT Utrecht, The Netherlands; 5 Department of Microbiology, University of Chicago, Chicago, USA

## Abstract

**Background:**

The Gram-positive bacterium *Enterococcus faecium *is an important cause of nosocomial infections in immunocompromized patients.

**Results:**

We present a pyrosequencing-based comparative genome analysis of seven *E. faecium *strains that were isolated from various sources. In the genomes of clinical isolates several antibiotic resistance genes were identified, including the *vanA *transposon that confers resistance to vancomycin in two strains. A functional comparison between *E. faecium *and the related opportunistic pathogen *E. faecalis *based on differences in the presence of protein families, revealed divergence in plant carbohydrate metabolic pathways and oxidative stress defense mechanisms. The *E. faecium *pan-genome was estimated to be essentially unlimited in size, indicating that *E. faecium *can efficiently acquire and incorporate exogenous DNA in its gene pool. One of the most prominent sources of genomic diversity consists of bacteriophages that have integrated in the genome. The CRISPR-Cas system, which contributes to immunity against bacteriophage infection in prokaryotes, is not present in the sequenced strains. Three sequenced isolates carry the *esp *gene, which is involved in urinary tract infections and biofilm formation. The *esp *gene is located on a large pathogenicity island (PAI), which is between 64 and 104 kb in size. Conjugation experiments showed that the entire *esp *PAI can be transferred horizontally and inserts in a site-specific manner.

**Conclusions:**

Genes involved in environmental persistence, colonization and virulence can easily be aquired by *E. faecium*. This will make the development of successful treatment strategies targeted against this organism a challenge for years to come.

## Background

Enterococci are Gram-positive commensals of the gastrointestinal tract of humans and other mammals. Currently, more than 30 species in the genus *Enterococcus *have been described [1]. The two most studied enterococcal species are *E. faecium *and *E. faecalis*, which are genetically distinct as shown by their 16S rRNA sequences and the sequences of the housekeeping gene *atpA *[2]. Both *E. faecium *and *E. faecalis *are an important cause of infections in hospitalized, immunocompromized patients [3]. Historically, *E. faecalis *has caused 90% of all enterococcal infections, but since the late 1980s a rapid increase in nosocomial *E. faecium *infections has occurred [4,5]. This expansion of *E. faecium *in the hospital environment coincided with the swift acquisition of multiple resistance mechanisms against many antibiotics. Resistance to ampicillin and vancomycin are probably the most significant resistance traits that have been acquired by *E. faecium *since the 1980s and nowadays the majority of *E. faecium *clinical isolates are resistant to these antibiotics. In contrast with *E. faecium*, resistance to ampicillin and vancomycin in *E. faecalis *is still a relatively rare trait [6,7]. Treatment of infections caused by antibiotic-resistant *E. faecium *is currently a clinical challenge and the few remaining therapeutic options are jeopardized by the emergence of resistance to new classes of antibiotics [8,9]. Therefore antibiotic-resistant *E. faecium*, together with methicillin-resistant *Staphylococcus aureus*, is currently seen as one of the gravest threats to successful therapy presented by Gram-positive bacteria [10].

Several studies have shown that the *E. faecium *strains that cause infections in hospitalized patients are different from the strains that colonize the gastro-intestinal tract of the healthy human host [11-13]. Population biology-based studies using multilocus sequence typing (MLST) suggested that the large majority of strains isolated from nosocomial infections belong to a distinct genetic lineage termed Clonal Complex 17 (CC17) [14,15]. In a previous study, in which comparative genome hybridization was used to assay diversity in *E. faecium*, more than 100 genes were found to be enriched in *E. faecium *CC17 isolates. Insertion sequence (IS) elements were the most prominent group of genes enriched in CC17 but, in addition, genes with a proven or proposed role in *E. faecium *virulence were also specifically identified in CC17 isolates [11].

Only two genes in *E. faecium *have been experimentally verified to contribute to virulence in animal models. These are the large surface protein Esp, which has a role in biofilm formation and urinary tract infection [16,17] and the MSCRAMM (Microbial Surface Components Recognizing Adhesive Matrix Molecules) Acm, which is an adhesin of collagen and contributes to endocarditis [18,19]. The *hyl *gene, which was proposed to encode a protein functioning as a hyaluronidase, is primarily found in clinical isolates and is much less common in *E. faecium *strains from other sources [20]. Although a *hyl *deletion mutant has not been described, the conjugative transfer of a plasmid containing the *hyl *gene made the transconjugant more virulent in a mouse peritonitis model, suggesting a role for the *hyl *gene in virulence [21]. In addition, binding to extra-cellular matrix components of a number of other surface proteins has also been shown experimentally and these proteins may contribute to virulence of *E. faecium *by acting as adhesins [22-24]. However, animal experiments of deletion mutants in the genes encoding these proteins need to be performed to further characterize the role (if any) of these proteins in *E. faecium *pathogenesis. The acquisition of genes conferring an infectious phenotype to *E. faecium *is thought to be a recent phenomenon, and coincides with the global emergence of *E. faecium *as a nosocomial pathogen since the late 1980s [15].

Molecular studies into the biology of *E. faecium *have so far been hampered by the poor genetic accessibility of this organism. Additionally, there has long been a marked lack of genome sequence information for *E. faecium *and consequently there exists a need for a multi-strain genomic analysis of *E. faecium *with the aim to describe its fundamental biology and to determine the causes of its emergence as a nosocomial pathogen.

In recent years, alternatives to Sanger-based genome shot-gun sequencing have been developed. These 'next-generation' sequencing technologies are characterized by dramatically higher throughputs and lower costs for sequencing than Sanger-based sequencing methodologies. Pyrosequencing, which is also known as 454 sequencing in reference to the company 454 Life Sciences (Branford, CT, USA) that markets pyrosequencing platforms, currently appears to be the most widely used next-generation sequencing method for *de novo *microbial genome sequencing [25]. While next-generation genome sequencing methods can be used to rapidly sequence a genome to completion in cases when no or very few repetitive elements are present [26], a more typical outcome is a draft genome assembly consisting of relatively large contigs which essentially cover the entire genome, but where gaps are caused by repetitive elements that are allocated to, generally small, separate contigs [27]. Pyrosequencing-based genome analyses of several micro-organisms of medical importance have recently been performed and have demonstrated the contribution of genomic islands for the development of pathogenicity and the large genetic diversity that can be contained within a single bacterial species [27-29].

In this study we use pyrosequencing-based genome sequencing of seven *E. faecium *strains to quantify the genomic diversity and determine core- and pan-genome size and to identify genomic differences between *E. faecium *and *E. faecalis*. The genome analysis of *E. faecium *also led to the identification of a large pathogenicity island that is associated with the *esp *gene. This study will provide a starting point for genome-wide studies to develop targeted strategies against this emerging nosocomial pathogen.

## Results

### Genome sequencing of seven *E. faecium *isolates

Four of the sequenced strains (E1162, E1636, E1679, and U0317) originated from human infections and consequently are referred to collectively as infectious isolates in this study. Strains E980 and E1039 were isolated from the faeces of healthy human volunteers. Strain E1071 was isolated as part of a hospital surveillance program in a Dutch hospital. This surveillance program was instigated because of an ongoing outbreak of vancomycin-resistant enterococci in the hospitalized patients. E1071 was isolated from a patient not suffering from an enterococcal infection and subsequent typing of the isolate showed that it was different from the strain causing the outbreak.

Prior to genome sequencing, MLST was used to determine the sequence type (ST) of the selected isolates. MLST is currently widely used for population studies of *E. faecium *and is based on DNA sequence analysis of seven housekeeping genes [30]. MLST of the seven selected isolates showed that they all had different STs and represented a sufficiently diverse sample of the species *E. faecium*. Two of the sequenced strains (E1162 and U0317) were assigned to CC17. The sequenced strains were chosen to cover a large period of time, with the most ancient strain being from the year 1961 (E1636) and the most recent from 2005 (U0317). The relevant characteristics of the sequenced isolates are summarized in Table 1.

**Table 1:** Origins of the strains of which the genomes were sequenced.

Strain	Source	MLST sequence type	CC17	Year of isolation	Country^*a*^
E980	Faecal isolate collected during community surveillance program [106], non-hospitalized person.	94	No	1998	NLD
E1039	Faecal isolate collected during community surveillance program [106], non-hospitalized person.	42	No	1998	NLD
E1071	Faecal isolate. Hospital surveillance program, hospitalized patient.	32	No	2000	NLD
E1162	Clinical isolate (bloodstream infection).	17	Yes	1997	FRA
E1636	Clinical isolate (bloodstream infection).	106	No	1961	NLD
E1679	Clinical isolate from a vascular catheter.	114	No	1998	BRA
U0317	Clinical isolate (urinary tract infection).	78	Yes	2005	NLD

^*a *^Country abbreviations. NLD: The Netherlands; FRA: France; BRA: Brazil.

The genomes of the seven selected *E. faecium *strains were sequenced using pyrosequencing technology [27] on the GS-FLX platform. The *E. faecium *genome sequence data obtained in this project are summarized in Table 2. Lander-Waterman statistics [31] estimate that more than 99.9% of each genome was sequenced. The genome sequences revealed roughly identical %G+C content, average gene size and %protein coding DNA for all strains. The quality of the generated sequences was evaluated using the Phred-like quality scores that are linked to these reads. Only between 0.027% and 0.196% of bases had quality scores lower than 20, which represents a small chance (1 in 100) for a mis-called base [27]. Thus, we conclude that these draft genome sequences are of good quality and can be used for further analyses. There is considerable variation in the number of assembled bases between the seven genomes, suggesting large differences in the size of the chromosome between strains and/or the variable presence of large plasmids. To determine the presence of large plasmids in the seven isolates of which the genome was sequenced we performed pulsed-field gel electrophoresis (PFGE) with S1 nuclease digestion of whole genomic DNA (Additional file 1). This revealed that in all isolates plasmids with sizes >50 kb are present. Four of the sequenced strains (E980, E1636, E1679 and U0317) have mega-plasmids that range in size between 200 and 240 kb. The variable presence of large plasmids between the isolates is therefore an important cause of the variation in number of assembled bases that was observed in these genome projects.

**Table 2:** General features of the *E. faecium *genomes.

Features	E980	E1039	E1071	E1162	E1636	E1679	U0317
Coverage of sequence	19.1×	24.4×	19.9×	20.0×	19.3×	16.7×	17.0×
Average sequence read length	240.0	246.0	254.3	256.7	237.6	239.8	235.6
Number of contigs	131	124	96	139	223	340	227
Number of assembled bases	2792626	2503230	2700770	2711396	2838335	2928184	2893029
Contig N50 (bp)	52826	46767	91386	50721	38772	20200	31583
Largest contig (bp)	145466	151666	228099	153370	152616	150593	162623
G + C content, %	38.1	38.0	37.9	38.0	37.8	37.7	37.7
Protein coding DNA, %	86.4	85.0	85.9	86.3	85.9	85.7	85.8
Number of predicted CDS	2869	2587	2714	2694	2940	3043	2965
Average gene length	841	822	855	869	830	824	837
Bases with quality score <20, %	0.051	0.035	0.027	0.034	0.104	0.196	0.155

Functional categorization by COG (Cluster of Orthologous Groups [32]) classification of the predicted proteome of the sequenced strains (Additional files 2 and 3) revealed a largely identical functional repertoire in the seven sequenced strains with the largest variance between strains in the percentage of proteins assigned to COG functional category L (replication, recombination and repair).

### Identification of antibiotic resistance determinants in the sequenced isolates

We determined antibiotic resistance profiles of the sequenced isolates by broth microdilution and linked the observed resistances to the presence of resistance genes (Additional file 4). These data show that strains that have been isolated recently from hospitals (either from patients that were colonized by *E. faecium *or suffered from *E. faecium *infections) are multi-resistant, while the human commensal strains E980 and E1039 have not acquired broad resistance to antibiotics. Practically all observed antibiotic resistant phenotypes could be linked to the presence of one or more different antibiotic resistance genes, most of which appear to reside on plasmids as can be concluded from the presence of plasmid replication or toxin/anti-toxin genes for plasmid maintenance on the same contigs as the antibiotic resistance genes. Two strains (E1071 and E1679) were found to be resistant to vancomycin. Both these strains carry the *vanA *transposon. In strain E1071 the *vanA *transposon is of the A2 type [33], in which a copy of an IS*1216V*-IS*3 *like element has inserted at the left end of the *vanA *transposon, which results in the deletion of the first 120 bp as compared to the archetypal Tn*1546 *vancomycin resistance element described by Arthur *et al*. [34]. In addition, a single nucleotide polymorphism (SNP) was identified in the *vanX *gene at position 8234 (G→T) of the *vanA *transposon, resulting in the substitution of a lysine to an asparagine in VanX. In strain E1679 the *vanA *transposon is essentially identical to the Tn*1546 *element [34] with only a single SNP (G→A) occuring at position 5663 resulting in the substitution of an alanine by a threonine in the VanS protein.

In addition, strains E1162, E1679 and U0317 are high-level resistant (minimum inhibitory concentration (MIC) >200 μg/ml) to ampicillin and have point mutations that are known to confer resistance to this antibiotic [35] in the *pbp5 *gene. Strain E1636, which is low-level resistant to ampicillin (MIC = 25 μg/ml), does not have these mutations. Presumably, other mechanisms, such as an elevated expression of *pbp5 *[36] could play a role here. The two strains (E1679 and U0317) that are resistant to ciprofloxacin both carry point mutations in the *gyrA *and *parC *genes, which have previously been described to be associated with resistance to ciprofloxacin [37].

On the basis of MLST, the multi-drug resistant strain E1071 groups with isolates from piggeries and healthy humans in a clonal complex that is generally not linked to human infections [15,38,39]. Genome analysis of E1071 suggests a possible pig origin of this strain on the basis of two observations. Firstly, E1071 was found to contain the *tcr *gene cluster, which confers resistance to copper [40]. Another putative copper detoxification system (EfmE1071_1339 to EfmE1071_1341) was also identified in E1071. This finding suggests a porcine origin of E1071 as resistance to copper in *E. faecium *is selected for in pigs because this metal is used as a growth-promoting feed supplement [41]. Secondly, the base pair found at position 8234 of the *vanA *transposon also suggests a porcine origin of E1071. The observed nucleotide T at position 8234 is associated with porcine isolates while a G at this position would indicate a strain originating from poultry [42]. Strain E1071 may therefore be representative of *E. faecium *strains that can be easily transferred between pigs and humans and may therefore shuttle antibiotic resistance genes between these niches.

### COG-based functional comparison between *E. faecium *and *E. faecalis*

A more detailed COG-based functional comparison between *E. faecium *and *E. faecalis *was performed to identify characteristics that distinguish these two species. A total of 70 COGs (Additional file 5) were present in all of the *E. faecium *genomes sequenced in this study, while being absent in six publicly available *E. faecalis *genome sequences. The reverse analysis, in which COGs were identified that are present in all *E. faecalis *genomes and absent from *E. faecium*, showed that 140 COGs are specific for *E. faecalis *(Additional file 6).

The COG-based comparison of *E. faecium *and *E. faecalis *revealed important differences between both organisms in sugar metabolism, particularly of the pentose sugar arabinose. The ability to use arabinose by *E. faecium *can be used to distinguish *E. faecium *from *E. faecalis *[43] but further characterization of the metabolism of this sugar in *E. faecium *has not been performed. In other lactic acid bacteria [44], the isomerization of arabinose to ribulose is the first step in the metabolism of this sugar. This reaction is predicted to be catalyzed by the *E. faecium *protein belonging to COG2160, which is not present in *E. faecalis*. Subsequently ribulose can be phosphorylated, after which the resulting intermediate ribulose-5-phosphate is further metabolized through the phosphoketolase pathway in which phosphoketolase (COG3957) is responsible for the crucial conversion of xylulose-5-phosphate to glyceraldehyde 3-phosphate (GAP) and acetyl-phosphate [44]. GAP is then further used as a substrate in the Embden-Meyerhof pathway. In *E. faecalis *proteins belonging to both COG2160 and COG3957 are absent and this probably explains the inability of *E. faecalis *to ferment arabinose. *E. faecium *also has COGs dedicated to the uptake of arabinose (COG4213, COG4214) and the degradation of arabinose-containing oligo-saccharides (COG3940), all of which are absent from *E. faecalis*.

The presence of COGs in *E. faecium *that are specifically involved in the metabolism of arabinose suggests that *E. faecium *has adapted its metabolism to include plant polysaccharides or their degradation products as energy sources since arabinose occurs abundantly in nature as subunits of hemicellulose. The ability of *E. faecium *to utilize carbon sources from plant origin is also exemplified by the presence in all *E. faecium *strains of proteins belonging to COG5424, which is predicted to be involved in the metabolism of pectin or its degradation products, and to COG3479, which is likely to be involved in the breakdown of coumaric acid and other components of lignocellulose as has been shown for a similar protein (81% amino acid identity to the *E. faecium *ortholog) in *Lactobacillus plantarum *[45]. Again, none of these COGs are present in *E. faecalis*. In contrast, a number of COGs that are unique to *E. faecalis *are linked to its previously described ability to use ethanolamine as a carbon source during anaerobic growth in the presence of cobalamin [46]. Ethanolamine is a common head group of phospholipids and is therefore abundantly present in the gut as part of the host's diet.

Another metabolic difference between *E. faecium *and *E. faecalis *was predicted in the first step of gluconeogenesis from pyruvate, i.e. the conversion of pyruvate into phosphoenolpyruvate (PEP). In *E. faecium*, pyruvate can be converted by pyruvate carboxylase to oxaloacetate and subsequently PEP is formed from oxaloacetate by the action of phosphoenolpyruvate carboxykinase (COG1866). In *E. faecalis*, the latter enzyme is not present and PEP is formed directly from pyruvate by the enzyme pyruvate phosphate dikinase (COG0574).

The COG-based comparison of *E. faecium *and *E. faecalis *also revealed differences in proteins involved in the protection against oxidative stress. In contrast to *E. faecalis *[47], *E. faecium *does not have the enzyme catalase, but it has other mechanisms putatively targeted at oxygen detoxification. One of these mechanisms is a peroxiredoxin and a corresponding reductase (COG3634). While *E. faecalis *also has proteins with a similar function (EF2738-EF2739 in the V583 genome [48]), the *E. faecium *proteins are not orthologous to these *E. faecalis *proteins, but instead are related to a similar and biochemically characterized system in *Thermus aquaticus *with 81% and 61% amino acid identity for the peroxiredoxin and its reductase, respectively [49]. Another possible defense mechanism to oxidative stress is provided by glutathione (γ-GluCysGly; GSH), which can be synthesized by both *E. faecium *and *E. faecalis *[50]. The reduced form of glutathione, together with the enzyme gluthatione peroxidase, can detoxify reactive oxygen species, resulting in the formation of GSSG [51]. *E. faecium *has a glutathione peroxidase (COG0386), but *E. faecalis *does not have this particular enzyme, suggesting that in *E. faecium *glutathione may play a more prominent role in the oxidative stress response than in *E. faecalis*. In contrast to *E. faecium*, *E. faecalis *is capable to respire aerobically in the presence of heme due to a cytochrome *bd*-type respiratory oxidase (COG1271 and COG1294; [52]), which leads to the production of substantial amounts of reactive oxygen species [53]. This particular selective pressure may have led to divergent evolution of the oxidative stress response in *E. faecium *and *E. faecalis*.

### Phylogenomic and diversity analysis of the *E. faecium *genome

**Figure 1:**
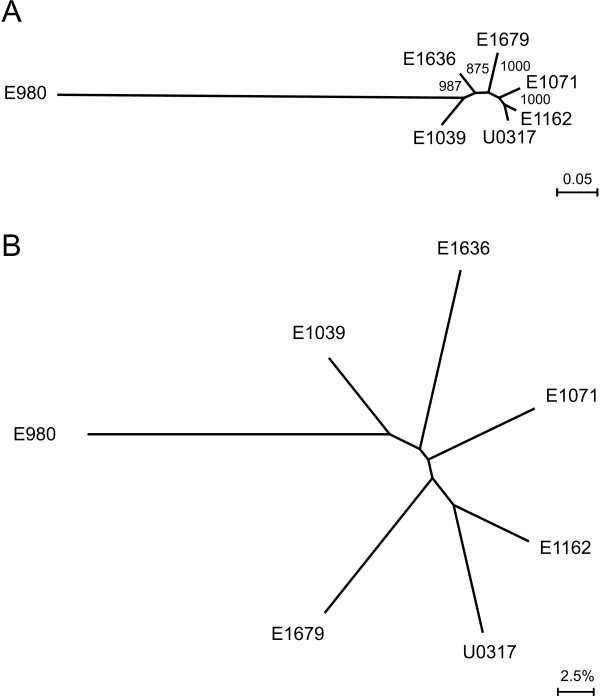
**Phylogenomic analysis of *E. faecium***. Panel (A): Unrooted maximum likelihood tree of *E. faecium *based on the concatenated alignments of 649 orthologous proteins (containing 11639 residues). Bootstrap values are based on 1000 permutations. Panel (B): Unrooted neighbour-joining tree depicting differences in gene content in *E. faecium*. The distance matrix used to generate this phylogenomic tree is provided as Additional file 7.

Phylogenomic trees for the seven *E. faecium *isolates were constructed based on concatenated alignments of 649 orthologous proteins in the genomes (Fig. 1A) and based on the overall gene content of the genomes (Fig. 1B). Fig. 1A is a maximum likelihood tree based on mutations in conserved proteins among the seven sequenced isolates and is therefore indicative of the evolutionary distance between the sequenced strains. Fig. 1B is a neighbor joining tree with shared gene content as distance measure (Additional file 7) and represents the differences between the strains caused by gene gain and loss. Both analyses resulted in phylogenomic trees of nearly congruent topology and demonstrate that strain E980 is evolutionary distinct from the other six sequenced isolates, which are more closely related to each other. While the evolutionary distance between these six isolates (as compared to E980) is relatively small, there are considerable differences in gene content between strains. This observation indicates that gene gain and loss, rather than evolutionary descent, appears to be the most important driving force in determining fitness of a given *E. faecium *strain in a specific environment. There is no evidence for a recent common ancestor of strains which have been isolated from an infection (E1162, E1636, E1679, and U0317). However, the two strains from CC17 (E1162 and U0317) appear to share a more recent common evolutionary history compared to the other isolates. While E1162 and U0317 appear to be relatively closely related to each other based on the alignment of orthologous proteins, there is a remarkably large difference (11.7%) in gene content between these two strains.

**Figure 2:**
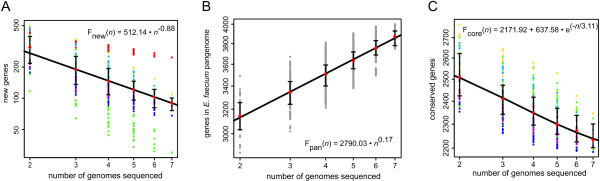
**Analysis of *E. faecium *genome diversity**. Estimates of new gene discovery rate (panel A), total *E. faecium *gene pool (pan-genome) (panel B) and core-genome size (panel C) are shown for increasing values of the number *n *of *E. faecium *genomes sequenced. Colored circles represent the number of new or core genes present when a particular genome is added to the subset of the remaining genomes. Grey circles represent the total gene repertoire for a random collection of organisms. Medians of the distributions are indicated by red diamonds. The curve for the estimation of the gene discovery rate is a least squares fit of the power law F_new_(*n*) = κ_new _*n*^-*α *^to medians. The curve for the estimation of the size of the *E. faecium *pan-genome is a least squares power law (F_pan_(*n*) = κ_pan _*n*^γ^) fit to the medians. The size of the core genome was estimated by fitting the exponential curve F_core_(*n*) = κ_core _exp[-*n*/τ_core_] + tg_core_(θ) to medians.

To further quantify the intra-species diversity of *E. faecium*, the coding sequences (CDS) predicted from the seven genomes were compared by a highly sensitive all-vs-all sequence alignment. Subsequently, the new gene discovery rate was estimated by fitting a least squares power law (F_new_(*n*) = κ_new _*n*^-*α*^) to the median number of new genes calculated for all strain combinations (Fig. 2A). The value for the exponent αdetermines whether the pan-genome can be considered to be essentially unlimited in size ("open"; α ≤ 1) or to have a finite size ("closed"; α > 1) [54]. In the case of *E. faecium*, the estimated value for α is 0.88 ± 0.02 signifying an open pan-genome and a high genomic diversity between *E. faecium *strains. To estimate the total size of the gene pool available to *E. faecium *(i.e., the *E. faecium *pan-genome), an analysis was performed using the same all-vs-all sequence alignment in which both genes that were shared and genes that are strain specific were counted. A least squares power law (F_pan_(*n*) = κ_pan _*n*^*γ*^) was fit to the medians and the resulting γ value of 0.17, being > 0, is again indicative of an open pan-genome [54] and confirms that new *E. faecium *genes will continue to be identified as more genomes are sequenced.

The size of the core genome was estimated by fitting an exponential curve to the median number of CDS conserved across all the genomes calculated for all strain combinations (Fig. 2B). The core genome size of *E. faecium *was estimated to be 2172 ± 20 CDS. This value shows that a sizeable fraction of the *E. faecium *genome is accessory. In E1039, which has the smallest genome in our data set, 16% of the genome is non-core, while this quantity increases to 29% in U0317.

### Bacteriophages in *E. faecium*

Analysis of the accessory genome of the *E. faecium *strains revealed that phage and phage-like sequences contribute significantly to the genomic diversity in *E. faecium *(Table 3). CDS of predicted phage origins make up between 2.3% (E1071) and 5.1% (E980) of the total number of CDS of the genome. In all isolates a significant fraction of these CDS are strain-specific, indicating the unique nature of the phage elements that are present in each genome. No CDS of phage origin were found to be specifically associated with infectious isolates. Clustered regularly interspaced short palindromic repeats (CRISPR) consist of short, conserved repeats interspaced by variable sequences and provide immunity against foreign genetic elements. CRISPRs have been identified in approximately 40% of sequenced eubacterial genomes and are associated with *cas *genes that are essential for the antiviral function of CRISPRs [55]. In three of the sequenced strains (E1071, E1679 and U0317) CRISPR-loci could be identified but in all strains the CRISPR locus is located inside a gene. The protein that is encoded by this gene is homologous to plasmid replication initiator proteins in other lactic acid bacteria. It seems unlikely that the gene's internal repeat sequences act as genuine CRISPRs. In addition, none of the sequenced *E. faecium *strains contain a homolog of the *cas1 *gene (COG1518), which is a universal marker for CRISPR-associated genes [55,56]. Thus, there is no evidence to suggest that a functional CRISPR-Cas system is present in the sequenced *E. faecium *strains and this may explain their diversity in prophage sequences. The fully sequenced *E. faecalis *V583 strain also does not contain the CRISPR-Cas system and its genome contains a number of prophage elements. These findings are in contrast to *E. faecalis *strain OG1RF [57], which has a CRISPR-locus and associated *cas *genes. Interestingly, the *E. faecalis *OG1RF genome does not contain prophage sequences [57].

**Table 3:** CDS of bacteriophage origin in the sequenced genomes

Strain	Number of CDS of bacteriophage origin^*a*^	Number of genome-specific CDS of bacteriophage origin^*a*^
E980	148 (5.2)	89 (3.1)
E1039	115 (4.4)	56 (2.2)
E1071	63 (2.3)	40 (1.5)
E1162	86 (3.2)	29 (1.1)
E1636	101 (3.4)	32 (1.1)
E1679	149 (4.9)	60 (2.0)
U0317	117 (3.9)	32 (1.1)

^*a *^Values in brackets denote the percentage of CDS relative to the total number of CDS in the genome. A CDS was scored as unique for a specific genome when the encoded protein did not have a BLAST-hit with 90% amino acid identity or higher in any of the other sequenced isolates.

**Figure 3:**
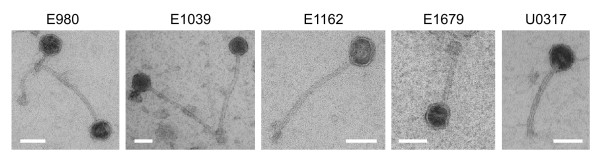
**Transmisssion electron micrographs of phage particles released by the indicated *E. faecium *strains upon overnight exposure of an exponentially growing culture to 1 μg/ml mitomycin**. Scale bars are 50 nm.

In five of the seven sequenced *E. faecium *strains phages could be activated by mitomycin through observation of phage particles by transmission electron microscopy of the culture supernatant (Fig. 3). All these phages had an isometric head with a long non-contractile segmented tail, which is characteristic of the *Siphoviridae*. *Siphoviridae *are a very common family of bacteriophages of the lactic acid bacteria [58,59]. While head-sizes are similar (~40 nm), there is marked variation in the size of the tails, ranging from ~70 nm in the E1679 phage to ~220 nm in the E1039 phage.

Phage lysates were tested for their infectivity but plaque formation was not observed (data not shown). This may be explained by a defect in infectivity of the phage particles, but it may also be possible that the prophages that are integrated into the different genomes protect the host against extra phage infection [60].

### Identification of potential and established virulence genes

To identify other genes potentially involved in virulence or the emergence of *E. faecium *as a nosocomial pathogen, the proteomes predicted to be encoded by the genomes of the strains that were isolated from human infections (E1162, E1636, E1679, and U0317) were compared to the predicted proteomes of the non-infectious isolates (E980, E1039 and E1071). This revealed that a group of 26 orthologous proteins are conserved in all infectious strains, while being absent in the non-infectious strains (Additional file 8). To estimate the relevance of this particular number of shared proteins, we performed a similar analysis to identify proteins shared between all possible four combinations of genomes, while being absent in the remaining three genomes. This analysis showed that the average number of shared proteins in a combination of four strains, while being absent in the three other strains is 3.7, with a median value of 2. The 26 shared proteins between infectious strains and absent from non-infectious strains represent the highest value for all possible combinations. Seven of the 26 proteins that occur solely in infectious isolates are IS elements of different families. These may contribute to the genomic flexibility in *E. faecium *and thereby facilitate the sequential acquisition of adaptive elements. In addition, an ABC transporter with unknown substrate specificity is also found to be specific for infectious isolates. Other proteins that are conserved in all infectious isolates have predicted roles in plasmid replication or have a putative function as phage type integrases/recombinases. Finally, a number of hypothetical proteins are only present in infectious isolates, two of which are predicted to be located in the cytoplasmic membrane.

The presence of the known virulence genes *esp*, *hyl *and *acm *in the sequenced isolates was also analyzed. The *esp *gene was found in strains E1162, E1679 and U0317. The *hyl *gene is only present in U0317. A full-length *acm *gene is present in E1162 and U0317. In other strains this gene is either inactivated by insertion of a transposon (E980 and E1071) or by mutations that introduce a premature stop-codon (E1039, E1636, and E1679). This finding is in line with previous research, which has shown that interrupted *acm *genes are common in strains not belonging to CC17 and that an intact *acm *gene may have contributed to the recent emergence of CC17 as a nosocomial pathogen [61].

### Identification and mobilization of an *E. faecium *pathogenicity island

**Figure 4:**
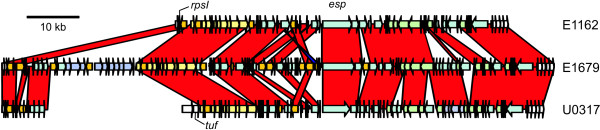
**Sequence alignment of the *esp *PAI in *E. faecium *E1162, E1679 and U0317**. The red-colored bands represent matching regions between the *esp *PAIs. A small blue band represents the inversion of a transposase fragment between *E. faecium *E1162 and E1679. Arrows indicate CDS and direction of transcription. The yellow arrows indicate the fragment of the *esp *PAI, that is identical to a fragment of the PAI of *E. faecalis *MMH594 [46]. The green arrows indicate the CDS that are homologous to EfaB5. The purple arrows in E1679 indicate the CDS predicted to encode the pathway of inositol metabolism. Orange arrows indicate transposases and integrases. White arrows indicate flanking genes that are not part of the *esp *PAI. The positions of the *rpsI*, *tuf *and *esp *genes are indicated.

Three sequenced strains (E1162, E1679, and U0317) harbor the *esp *gene, which encodes an approximately 200 kDa protein that is anchored to the peptidoglycan of the cell wall through the presence of an LPxTG-type motif. In *E. faecium*, Esp is involved in biofilm formation and urinary tract infection in a mouse model [16,17]. In a previous study, the *esp *gene was proposed to be carried on a pathogenicity island (PAI), but only 14 kb of the PAI was sequenced in a single strain and the integration site of the PAI was not resolved [62]. By using the genome sequences of the *esp*^+ ^strains and after additional sequencing of PCR products that were generated to span gaps between contigs, we were able to complete the sequence of the *esp *containing PAI for all three strains. This revealed that *esp *is carried on a large PAI in *E. faecium *that is considerably larger than the 14 kb of sequence that was previously obtained [62] as it varies in size between 64 kb (in E1162), 104 kb (in E1679) and 68 kb (in U0317) (Fig. 4). There is some variation in the %G+C-content of the *esp *PAI (35.7% %G+C in E1162; 36.6% in E1679; 35.4% in U0317). This is 1.1 to 2.3% lower than the average %G+C-content over the entire genome of these strains, indicating that the DNA of the *esp *PAI is acquired exogenously. In E1162 and E1679 the *esp *PAI is flanked by an imperfect 54-bp direct repeat (Additional file 9), with the left repeat being the 3' end of the *rpsI *gene (which codes for the ribosomal protein S9) and the right repeat just upstream of a small gene encoding a 9 kDa protein with unknown function. In U0317 the 3' end of the PAI is inserted at the same genomic location as in the other strains and is flanked by the repeat sequence, but the 5' end has integrated downstream of the *tuf *gene. The *rpsI *gene is present in this strain, but only a small element, which is identical to the 5' part of the E1679 *esp *PAI, is found downstream. This suggests that the complete *esp *PAI in U0317 has originally inserted downstream of *rpsI*, but that since then a genomic rearrangement event has occurred, which finally resulted in nearly the complete *esp *PAI reintegrating downstream of *tuf*.

The sequence of large parts of the *esp *PAI is conserved between the three strains and diversity within the *esp *PAI is mainly caused by the variable presence of genetic elements. The most striking insertion in the *esp *PAI has occurred in E1679 and consists of a 7-kb gene cluster near the 5' end of the PAI, which encodes a complete pathway for inositol metabolism. Of the seven sequenced isolates, only E1679 is capable of using inositol as a carbon source (data not shown), suggesting that the metabolic pathway encoded by this element is functional. In all strains, a 10-kb element is present in the 5' part of the PAI that is essentially completely identical (= 98% nucleotide identity) to a gene cluster (EF0093 - EF0106) in the 154-kb pathogenicity island of *E. faecalis *MMH594 [63]. Interestingly, this *E. faecalis *PAI also carries the *esp *gene. The presence of these regions of identical DNA in both *E. faecium *and *E. faecalis *PAIs suggests a recent transfer of genetic material between these two species or acquisition from a third source. The function of this gene cluster in both *E. faecium *and *E. faecalis *is unclear. Three CDS of the element are predicted to encode ribosomal proteins and among the other encoded proteins are a membrane protein putatively functioning as a multi-drug transporter and membrane proteins that appear to be involved in the uptake of manganese. Interestingly, manganese uptake systems have been linked to virulence in various Gram-negative and Gram-positive bacteria [64]. Another gene which can play a role in host-cell interactions is found on the extreme 3' end of the *esp *PAI. This gene encodes a 120 kDa LPxTG-type cell wall anchored protein that is 91% identical to the EcbA protein from *E. faecium *DO, which was shown to acts as an adhesin to the extracellular matrix components fibrinogen and collagen [24]. The 3' end of all three *esp *PAIs is closely related (90% nucleotide identity) to an element termed EfaB5 [65] in *E. faecalis *(encoded by EF1875 - EF1889 in *E. faecalis *V583). In its turn, the EfaB5 element is closely related to the conjugation module of Tn*916*.

**Figure 5:**
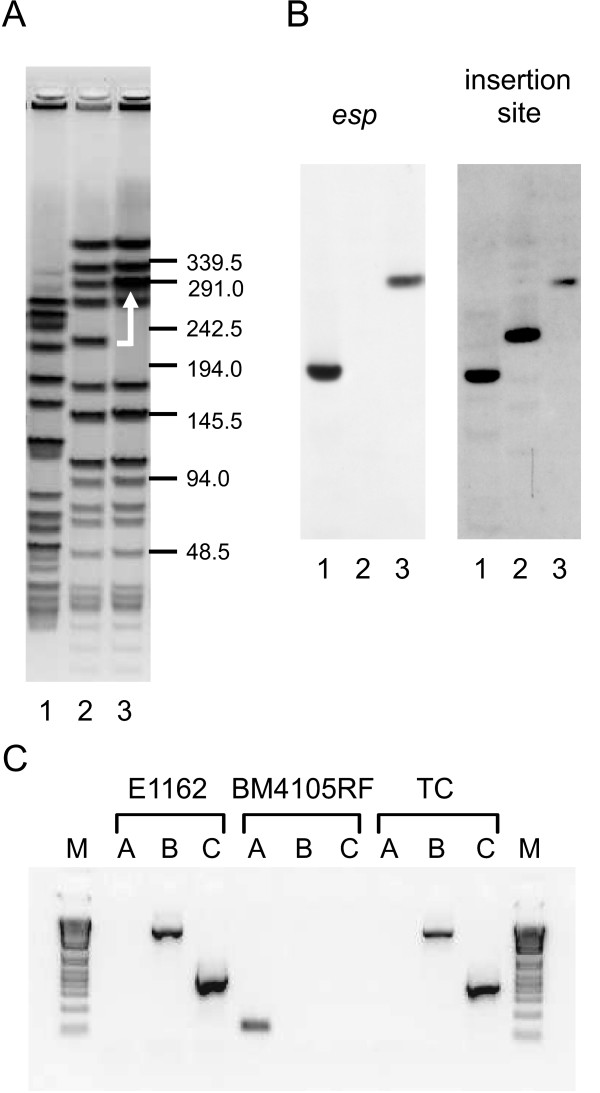
**Transfer of the *esp *PAI from *E. faecium *E1162Δ*esp *to *E. faecium *BM4105RF**. Panel (A): representative ethidium bromide stained PFGE gel of SmaI-digested chromosomal DNA of the donor strain (E1162Δ*esp*; lane 1), the recipient strain (BM4105RF; lane 2) and a transconjugant (lane 3). The gel band that has shifted in the recipient strain due to the insertion of the *esp *PAI is indicated by the white arrow. Panel (B): Southern blots of the PFGE gel hybridized with a probe for *esp *and a probe covering the insertion site of the *esp *PAI. Panel (C): PCR analysis of the *esp *PAI insertion site in the donor strain (E1162Δ*esp*), the recipient strain (BM4105RF) and a transconjugant (TC). PCRs were performed with primers covering the *esp *PAI insertion site (reactions A), covering the 5' end of the *esp *PAI and the 5' flanking end of the insertion site (reactions B), and covering the 3' end of the *esp *PAI and the 3' flanking end of the insertion site (reactions C). The marker (M) is Invitrogen's 1 Kb Plus DNA Ladder. Note that, because of the size of the *esp *PAI, no products can be obtained in PCRs with primers on the 5' and 3' flanking regions of the *esp *PAI integration site in *esp*^+ ^strains.

Previously, the *esp *gene was shown to be transferable by conjugation between two *E. faecium *isolates [66] but no further analysis of the transfer of the *esp *gene was performed in this study. Our observation that *esp *is located on a PAI led us to reinvestigate the mobilization of *esp*, to assess if the *esp *PAI is completely or partially transferred and if the *esp *PAI inserts in a site-specific manner. We performed filter mating experiments with *E. faecium *E1162Δ*esp *(a derivative of E1162 with a chloramphenicol resistance cassette inserted in the *esp *gene [16]) as donor strain and BM4105RF as recipient. Transconjugants were picked up with a frequency of 1.6 × 10^-7 ^per recipient cell. Insertion of the PAI in the recipient strains was confirmed by pulsed-field gel electrophoresis (PFGE) (Fig. 5A) with subsequent Southern blotting with a probe for the non-deleted part of *esp *(Fig. 5B). This showed that a single fragment in the transconjugant had increased ~60 kb in size and hybridized with the *esp *probe, which corresponds to transfer of the complete *esp *PAI of E1162. To determine the insertion site of the *esp *PAI in the transconjugants, we also probed the blot with a PCR fragment covering the putative integration site and found an identical ~60 kb increase in size of the hybridizing band, showing that the *esp *PAI has inserted at the same chromosomal location (i.e. downstream of the *rpsI *gene) as in E1162. These data were also confirmed by performing PCRs with primers that annealed to the flanking regions of the *esp *PAI and the 5' and 3' ends of the *esp *PAI (Fig. 5C) and subsequent sequencing of the PCR products (data not shown). Our data show that the complete PAI can be transferred from an *esp *PAI carrying strain to an *esp*^- ^strain and inserts in a site-specific manner directly downstream of the *rpsI *gene. Moreover, due to the use of an *esp *deletion mutant as donor in the conjugation experiment, we can conclude that the Esp protein is not essential for this process.

Remarkably, in the strains E980, E1071 and E1636, which do not harbor the *esp *PAI, other elements have inserted in the same locus, suggesting that this is an important hot-spot for integration and recombination in *E. faecium*. These elements are considerably smaller than the *esp *PAI. In E980, a 9-kb element encodes a phosphotransferase (PTS) system and a glycoside hydrolase (family 38). In E980 a deletion of 5 genes that form the right flanking region of the *esp *PAI has occurred. In both E1071 and E1636, different elements (both 8 kb in size) have inserted into the putative hot spot. They encode no obvious functions, but the presence of two distinct *repA*-like genes indicates that these elements are integrated plasmids. In both E1071 and E1636 the inserted elements are flanked by the 54-bp direct repeat (Additional file 9).

## Discussion

In the last two decades *E. faecium *has emerged as a nosocomial pathogen in severely immunocompromized patients. Successful treatment is increasingly hampered by antibiotic resistance, and consequently, *E. faecium *infections are associated with considerable morbidity and high mortality [10]. To obtain a better understanding of the basic biology of *E. faecium *and to estimate the diversity contained within this species we sequenced the genomes of seven *E. faecium *strains that were isolated from different ecological niches. This is the first study which employs genome sequencing for the study of this important nosocomial pathogen.

Resistance to several antibiotics was observed in all sequenced isolates and these phenotypically observed resistances could generally be linked to the presence of previously described resistance determinants in the isolates. Isolates from clinical sites were particularly rich in antibiotic resistance genes. The association of the antibiotic resistance genes with mobile genetic elements that we observed in the seven isolates under study here is in line with previous studies [67] and suggests that *E. faecium *may serve as an important hub for the transfer of antibiotic resistance genes to other intestinal bacteria [68,69].

A comparison between *E. faecium *and *E. faecalis *revealed differences in the occurence of several protein families between the two organisms. In particular, *E. faecium *has several metabolic pathways dedicated to the metabolism of carbohydrates of plant origin, which are absent from *E. faecalis*. This suggests an evolutionary adaptation to a lifestyle as a gut symbiont of herbivorous or omnivorous animals and may also enable *E. faecium *to survive on plant surfaces from which it can frequently be isolated [70]. The proteins tentatively involved in the protection against oxidative stress in *E. faecium *are very different from those in *E. faecalis*. The oxidative stress response of *E. faecalis *has been characterized in considerable detail and appears to be linked to virulence [71]. Interestingly, exogenous production of hydrogen peroxide by *E. faecium *has been identified as an important virulence factor in a *Caenorhabditis elegans *model of infection [72]. Understanding how *E. faecium *can protect itself from the reactive oxygen species that it produces itself or to which it is exposed to in neutrophils or macrophages during infection, may provide important insights into the process by which *E. faecium *can cause disease.

This study also provides novel insights into the population structure of *E. faecium*, which has so far been defined by MLST. Upon analysis of the MLST data using the algorithm eBURST, it has been suggested that the large majority of strains isolated from nosocomial infections group in a distinct cluster, termed CC17 [14]. However, the use of MLST in combination with the eBURST-algorithm to describe the population structure of *E. faecium *has been disputed as high recombination rates in *E. faecium*, in combination with a relatively low mutation rate, results in unreliable phylogenetic inferences by eBURST [73]. Recently, an analysis of the MLST database for *E. faecium *using novel and more advanced algorithms than eBURST has suggested that the STs constituting CC17 have in fact evolved independently from different ancestral clones [74]. The phylogenomic analysis presented here (Fig. 1) provides a detailed insight into the evolutionary descent of the seven sequenced strains and can be used to further refine our understanding of the evolution of *E. faecium*. In this study, two of the seven sequenced isolates (E1162 and U0317) have been assigned to CC17 on the basis of MLST. Our genomic data, combined with the recent insights obtained by MLST, suggest that the CC17 isolates appear to have formed a sub-population in the *E. faecium *species. However, it is also clear that these infectious isolates are not strictly clonally related to each other and have diversified considerably. In addition, the large differences in gene content observed here show that even in strains that are relatively closely related on the level of their core genome, the gain and/or loss of mobile genetic elements is the leading force in determining strain-specific properties.

The pan-genome analysis of *E. faecium *indicated that the total available gene pool within this species is essentially unlimited. This may be explained by its ability to incorporate foreign DNA into its gene pool and by the wide variety of ecological niches, such as the mammalian gastrointestinal tract and foods, that *E. faecium *can colonize. This allows the organism to come into contact with many pathogenic and non-pathogenic bacteria and sets the scene for extensive horizontal gene transfer between *E. faecium *and others. A consequence of the open pan-genome of *E. faecium *is that it can rapidly acquire genes that have the potential to increase fitness under adverse environmental conditions. This is most obvious in the accumulation of antibiotic resistance genes in clinical isolates (Additional file 4). In addition, genes that allow for infection of the immunocompromized host may also be acquired and introduced into the *E. faecium *gene pool, as is exemplified by the *esp *PAI.

Lysogenic bacteriophages have so far not been studied in *E. faecium*, but they make an important contribution to the genomic diversity of *E. faecium*. It has been hypothesized that lysogenic bacteriophages should confer some advantage to the host, because otherwise they would be lost due to competition with non-infected strains. They may also increase the environmental fitness of the lysogenic host in a process termed lysogenic conversion, which has been described to add to the virulence of several Gram-positive pathogens such as *Streptococcus pyogenes *and *S. aureus *[60]. It remains to be determined to what extent bacteriophages contribute to niche adaptation and an infectious phenotype in *E. faecium*, but the wide variety of phages that has been identified in this relatively small sample of strains show that they have a major role in shaping the genome of *E. faecium*. The absence of a functional CRISPR-Cas system in the sequenced *E. faecium *isolates may make these strains relatively susceptible to phage attack, resulting in a high diversity of integrated prophages. The CRISPR-Cas system has also been implicated in the spread of antibiotic resistance genes among staphylococci [75]. It is currently unknown if the CRISPR-Cas system also acts as a barrier to the acquisition of antibiotic resistance genes in enterococci.

The *esp *gene of *E. faecium *is more abundant in clinical isolates than in isolates from food or environmental origins [76,77] and contributes to biofilm formation and urinary tract infections in an animal model [16,17]. Here we show that *esp *is carried on a large pathogenicity island. Also in *E. faecalis*, the *esp *gene is harbored on a large pathogenicity island of approximately 150 kb in size. This *E. faecalis *PAI also carries the genes needed for the production of cytolysin, an exotoxin that contributes to *E. faecalis *virulence [64]. In *E. faecium *we have not observed a homolog of the *E. faecalis *cytolysin and in fact most other genes of the *E. faecalis *pathogenicity island as described in strain MMH594 [63] are absent in the *E. faecium esp *PAI. The exceptions to this rule are the *esp *gene itself and the 10-kb gene cluster described above, which is completely conserved between the *esp *PAIs of *E. faecium *and *E. faecalis*. The presence of this conserved element suggests recent horizontal transfer of these genes between *E. faecium *and *E. faecalis *or, possibly, the independent acquisition of this element by *E. faecium *and *E. faecalis *from another common source. Horizontal transfer may also explain the presence of the EfaB5 element on the 3' end of the *esp *PAI in *E. faecium*. This element is part of a larger family of conjugative and integrative elements in many Gram-positive bacteria. Exchange of specific modules in these elements appears to be a relatively common event. The *esp *PAI may, therefore, be the result of the serendipitous accumulation of several genetic elements in the hot spot provided by the *rpsI *gene in the *E. faecium *genome. Interestingly, also in *Streptococcus agalactiae *several mobile genetic elements have been found to be integrated downstream of *rpsI *[78], suggesting that this gene forms a hot spot for genomic diversity in different Gram-positive bacteria.

Even though the overall architecture of the *esp *PAI is identical in all sequenced isolates, there are also marked differences between the *esp *PAIs in the three different isolates described in this manuscript. These are mainly caused by the independent acquisition of other elements, such as the putative inositol metabolic pathway in strain E1679. This shows that recombination in the *esp *PAI is an ongoing process. Because the *esp *PAI is mobilizable, as demonstrated here by the conjugative transfer of the PAI between the strains E1162Δ*esp *and BM4105RF, the *esp *gene and the other genes that are carried on the pathogenicity island may spread rapidly through *E. faecium*, thereby contributing to the ability to infect immunocompromized human hosts.

## Conclusions

In summary, we have performed the first genome-based study of the nosocomial pathogen *E. faecium*. We found that the isolates originating from the hospital have acquired multiple antibiotic resistance genes and, in addition, have genes that may play a role in the colonization and infection of hospitalized patients. Genome sequencing of multiple isolates allowed us to determine a genome-based phylogeny of *E. faecium *and to accurately quantify the substantial genomic diversity between strains in this species. This analysis showed that the two strains belonging to CC17 in our data-set were relatively closely related on the basis of their core genome but still have a large difference in gene content, suggesting that gain and loss of mobile genetic elements, rather than evolutionary descent, is the most important driving force in determining virulence-associated properties in this clonal complex. The variable presence of lysogenic bacteriophages in the sequenced strains was found to be an important contributor to the intra-species diversity of *E. faecium*. We also identified characteristics that distinguish *E. faecium *from the related nosocomial pathogen *E. faecalis*, particularly in the repertoire of genes involved in sugar metabolism and the response to oxidative stress. Finally we show that three isolates have acquired a large PAI that is associated with the *esp *gene and we provide evidence that horizontal gene transfer between *E. faecium *and *E. faecalis *may have occurred during the evolution of this PAI.

While *E. faecium *currently has a lower virulence potential than other cocci with a low-%G+C content, such as *S. aureus *and pathogenic streptococci, its resistance to antibiotics has made it one of the most difficult-to-treat nosocomial pathogens. The genomic flexibility of *E. faecium *allows the efficient integration of additional fitness determinants to the *E. faecium *gene pool, leading to the rapid adaptation to new environmental niches such as those that are found in hospitalized patients. Genome-wide studies, which will be facilitated by the sequence data presented here, are therefore needed to increase our understanding of the basic biology of *E. faecium *and to identify genes that are essential for colonization and infection of hospitalized patients.

## Methods

### Strains, growth conditions and DNA isolation

The origins of the *E. faecium *strains of which the genome was sequenced are presented in Table 1. Stocks for these strains were stored at -80°C in Brain Heart Infusion (BHI) broth with 30% glycerol. Freezer stocks were streaked on Tryptic Soy Agar supplemented with 5% sheep blood (Becton Dickinson, Alphen aan den Rijn, The Netherlands). Single colonies were picked to inoculate 5 ml BHI. These cultures were grown for 18 hours at 37°C with shaking at 150 rpm and subsequently cells were pelleted by centrifugation at 4000 *g *for 10 min. Genomic DNA was isolated from the cell pellets using the Wizard Genomic DNA Purification kit (Promega, Leiden, The Netherlands) according to the manufacturer's instructions. MLST was performed as previously described [30]. It should be noted that we did not separate chromosomal DNA from plasmid DNA in the DNA preparations that were submitted for sequencing as it has been reported that many important characteristics of *E. faecium *(such as antibiotic resistance and virulence genes) are encoded on plasmids [21,79-81]. Plasmid content of the seven isolates was determined by PFGE on S1 nuclease treated total DNA according to previously published methods [81]

### Genome sequencing and annotation

Genome sequence data were generated with the GS FLX system (454 Life Sciences, Branford CT). A library of single-stranded template DNA fragments was prepared from the purified genomic DNA using the GS FLX Standard DNA Library Preparation Kit (Roche, Almere, The Netherlands). The Shotgun GS FLX Standard emPCR Kit I (Roche) was used for emulsion-based clonal amplification of a single-stranded template DNA library. The GS FLX Standard LR70 Sequencing Kit (Roche) was used in combination with the GS FLX Standard PicoTiterPlate Kit (70 × 75; Roche) to determine the nucleotide sequence of the immobilized and clonally amplified DNA library. All kits were used according to the manufacturer's instructions. The obtained sequences were *de novo *assembled using Newbler (454 runAssembly software, version 1.1.02.15).

The contig sequences of each strain were concatenated with the sequences NNNNNCACACACTTAATTAATTAAGTGTGTGNNNNN, which puts stop codons in all six reading frames, and the concatenated DNA sequences were submitted to the Annotation Service of the J. Craig Venter Institute (JCVI; Rockville MD, USA) [82], where they were run through JCVI's prokaryotic annotation pipeline. Included in the pipeline is gene finding with Glimmer [83], Blast-extend-repraze searches [84], HMM searches [85-87], TMHMM searches [88], SignalP predictions [89] and automatic annotations from AutoAnnotate. The automated annotations produced by the JCVI Annotation Service were curated manually. rRNA genes in the genomes were identified using RNAmmer [90]. The whole genome sequence data described in this paper have been deposited under the following accession numbers: *Enterococcus faecium *E980: [Genbank:ABQA00000000]; *Enterococcus faecium *E1039: [Genbank:ACOS00000000]; *Enterococcus faecium *E1071: [Genbank:ABQI00000000];*Enterococcus faecium *E1162: [Genbank:ABQJ00000000]; *Enterococcus faecium *E1636: [Genbank:ABRY00000000]; *Enterococcus faecium *E1679: [Genbank:ABSC00000000]; *Enterococcus faecium *U0317: [Genbank:ABSW00000000].

The annotated proteins were assigned to Cluster of Orthologous Groups (COG) by performing a sensitive search using the Smith-Waterman algorithm against the COG database as described previously [91] with a cut-off of E < 0.01. For the COG-based comparison of *E. faecium *with *E. faecalis *the protein files corresponding to all the *E. faecalis *genome sequences (strains ATCC29200, HH22, TUSoD Ef11, TX0104, TX1332 and V583) that were available at the time of analysis were downloaded from the NCBI Genomes website and COGs were assigned as described above. CRISPR loci in the genome sequences were detected by CRISPRFinder [92]. The occurence of CRISPR-associated *cas *genes was determined by the presence of a protein belonging to COG1518, which serves as the universal marker of CRISPR-associated genes [38,39]. Metabolic pathway mappings were performed using the KEGG Automatic Annotation Server [66]. The NCBI BLAST package [93] was used to compare protein and DNA sequences. DNA sequence comparisons were visualized with the Artemis Comparison Tool (ACT [94]). ACT comparison files were generated through DoubleACT [95], with a cut-off score of 1 × 10^-10^.

### Construction of phylogenomic trees

A phylogenomic tree based on the concatenated alignment of conserved protein sequences was constructed. First, proteins were selected that fitted the following criteria: (i) proteins that could be assigned to a COG and were the only protein present in this COG, and (ii) the COGs containing a single protein should be present in all seven sequenced genomes. One COG was removed manually from this dataset because of large inter-strain variation in the protein sequences which is caused by the assignment of non-orthologous proteins to the same COG. The resulting protein sequences, belonging to 649 different COGs, were subsequently aligned using MUSCLE [96]. These alignments were concatenated after which a maximum likelihood tree was constructed using PHYML [97], including bootstrapping with 1000 iterations. The resulting tree was visualized in SplitsTree4 [98].

To generate a phylogenetic tree based on gene content, pair-wise BLAST comparisons of the proteins encoded for each possible genome pair were performed. Proteins-pairs were scored as conserved between two genomes when the two proteins were each other's best BLAST hits and had an identity of ≥ 90%. The distance between two genomes was then calculated as 1 - [number of conserved CDS/number of CDS in smallest genome]. Based on this resulting distance matrix, a neighbour-joining tree was constructed and visualized using SplitsTree4 [98].

### Determination of antibiotic resistance in *E. faecium*

The MICs of the antibiotics ampicillin, chloramphenicol, ciprofloxacin, erythromycin, gentamicin, spectinomycin, streptromycin, tetracycline, and vancomycin against the seven *E. faecium *strains of which the genomes were sequenced, was determined in a broth microdilution assay in cation-adjusted Muller-Hinton Broth (Oxoid, Basingstoke, United Kingdom) according to previously published methods [99,100]. Antibiotics were obtained from Sigma-Aldrich (Zwijndrecht, The Netherlands).

### Pan- and core-genome analysis

The new gene discovery rate, and the size of the pan- and core-genome were estimated for *E. faecium*. All-vs-all WU-BLASTP [101] and all-vs-all WU-TBLASTN searches were performed on the seven *E. faecium *genomes. Results from these two searches were combined such that WU-TBLASTN hits prevented missing gene predictions from producing false-negatives. Hits were filtered using 50% sequence similarity and 50% coverage, as previously described previously [102]. The number (N) of independent measurements of the core genes (those shared by all *E. faecium *isolates) and new genes present in the *n*^th ^genome is N = [S]!/[(*n *- 1)!· ([S] - *n*)!], where S = 7, the number of sequenced strains. To reduce the number of required computations 1,000 measurements were randomly sampled for each value of *n*. The new gene discovery rate was modeled by fitting the power law function F_new_(*n*) = κ_new _*n*^-*α *^to the median number of new genes calculated for all strain combinations where *n *is the number of strains, and *κ*_new _and α are free parameters. An α ≤ 1 indicates an open pan-genome while α > 1 indicates a closed pan-genome [54]. The pan-genome itself represents the trend of the complete gene repertoire as more genomes are sequenced. The median number of shared and new genes was calculated for all strain combinations and a power law F_pan_(*n*) = κ_pan _*nγ *where *n *is the number of strains and *κ*_new _and γ are free parameters. γ > 0 indicates an open pan-genome [54]. The number of core genes was estimated by fitting the exponential decay function F_core_(*n*) = κ_core _exp [-*n*/τ_core_] + tg_core_(θ) to the median number of core genes calculated for all strain combinations. *n *is the number of sequenced strains and κ_core_, τ_core_, tg_core_(θ) are free parameters. tg_core_(θ) represents the extrapolated number of core genes assuming a consistent sampling mechanism and a large number of completed sequences.

### Bacteriophages in *E. faecium*

A first group of putative CDS of bacteriophage origin were identified based on the annotation (as phage proteins or with "prophage functions" as predicted cellular role) provided by the JCVI Annotation Pipeline. The phage origins of these proteins were further confirmed by BLAST searches which resulted in the identification of homologous proteins of bacteriophages in Firmicutes. These analyses were then expanded to include genes that appeared to form part of a complete or partial phage genome on the basis of the presence of one or more prophage modules in a larger gene cluster.

The activation of bacteriophages in the sequenced *E. faecium *strains was studied by exposing *E. faecium *cultures in the exponential growth phase (A_660 _= 0.2) to 1 μg/ml mitomycin, followed by a further incubation for 18 h at 37°C. These phage lysates were centrifuged (4150 *g*, 5 min) and 6-ml filter-sterilized aliquots of the supernatant were then ultracentrifuged at 81,000 *g *for 2 h. Pellets were washed with 0.1 M ammonium acetate, pH 7.5, followed by another ultracentrifugation step (81,000 *g*, 2 h.) and resuspended in 100 μl 50 mM Tris-HCl, pH 7.5, 86 mM NaCl, 17 mM MgSO_4_. Transmission electron microscopy with negative staining using uranylacetate was performed on these samples as described previously [22].

Infectivity of the phages was tested by the soft-agar layer technique. BHI plates were covered with BHI with 4% bacteriological agar, which was inoculated at 0.5% with an overnight culture of an indicator strain. As indicators the seven strains of which the genome was sequenced in this study were used. After the soft agar layer had cooled down to room temperature, 10 μl aliquots of the filter-sterilized phage lysate was spotted on the plates. Subsequently, plates were incubated overnight at 37°C, followed by visual inspection for phage plaques.

### Identification and mobilization of the *E. faecium *esp PAI

To close the sequence of the contigs containing the *esp *PAI in E1162, E1679 and U0317 sequence alignments were generated with the genome sequences of strains that did not carry the *esp *gene and previously published sequence of the genes immediately flanking the *esp *gene [62]. This resulted in a putative order of contigs surrounding the *esp *PAI, which was then confirmed by designing primers (sequences available on request) on the contig ends, followed by PCR (using AccuPrime™ Taq DNA Polymerase High Fidelity; Invitrogen, Breda, The Netherlands) and Sanger-sequencing of the generated PCR products. Assembly of the *esp *PAIs based on the generated sequences of gap-spanning PCRs and the contig sequences was done using Seqman 4.0 (DNAStar, Madison WI).

The mobilization of the *esp *PAI was studied by performing filter-mating experiments with strain E1162Δ*esp *[16], which carries a chloramphenicol resistance cassette that is integrated in the *esp *gene, as donor strain and strain BM4105RF, a rifampicin and fusidic acid resistant derivative of the plasmid-free strain *E. faecium *BM4105 [103], as recipient strain. Overnight cultures of these strains were diluted 1:20 in BHI broth and further cultured at 37°C until A_660 _= 1.0. Subsequently, 1 ml aliquots of both cultures were mixed, spun down (2 min, 14,000 *g*), and after washing the cell pellet with phosphate buffered saline (PBS; 138 mM NaCl, 2.7 mM KCl, 140 mM Na_2_HPO_4_, 1.8 mM KH_2_PO_4_, adjusted to pH 7.4 with HCl), the cells were resuspended in 30 μl BHI. The cell suspension was spotted on a nitro-cellulose filter (25 mm diameter, 0.45 μm; VWR, Amsterdam, The Netherlands) that was placed on Tryptic Soy Agar supplemented with 5% sheep blood. After overnight incubation, the filter was resuspended in 1 ml BHI and serial dilutions of this cell suspension were prepared in PBS. Aliquots of 200 μl were spread on BHI plates containing chloramphenicol at 10 μg/ml (for quantification of the number of cells of the donor strain), on BHI plates containing rifampicin and fusidic acid, both at 25 μg/ml (for quantification of the number of cells of the recipient strain), and on BHI plates containing all three antibiotics, to isolate transconjugants. Transconjugation efficiency was calculated as the viable counts on the BHI plates with chloramphenicol, rifampicin and fusidic acid divided by the viable counts on BHI plates with rifampicin and fusidic acid. This assay was perfomed in triplicate. Potential transconjugants were tested for presence of the *esp *gene by PCR as described previously [104]. PFGE on SmaI-digested total DNA was performed as described previously [105] to confirm that the transconjugant had the same genetic background as *E. faecium *BM4105RF. Lamba Ladder PFG Marker (New England Biolabs, Ipswich MA) was used to estimate the sizes of the PFGE fragments. Southern blotting and probe hybridization was performed as described previously [16]. The probes used in the hybridizations were generated by PCR with the primer-pairs esp.14F (5'-AGATTTCATCTTTGATTCTTGG-3') and esp.12R (5'-AATTGATTCTTTAGCATCTGG-3'), and DO1.F (5'-GCTTACTTACTCGTGACGC-3') and DO1.R (5'-GAAAAGTTAGGATTAAGGTAACTGC-3') on chromosomal DNA of strains E1162 and DO, respectively. The *esp *PAI insertion site was also studied using PCRs with primer-pairs DO1.F and DO1.R, which cover the *esp *PAI insertion site, with primer pairs DO1.F and PAIup.R (5'-CAAACATTAAGCTTTTTCACTTC-3'), which cover the 5' end of the *esp *PAI and with primer pairs PAIdown.R (5'-CCAACAATTGCTTGAACAG-3') and DO1.R, which cover the 3' end of the *esp *PAI and the 3' flanking end of the insertion site. Due to sequence heterogeneity in the downstream region of the *rpsI *gene, the primer DO1.R did not yield a product in PCRs on BM4105RF and the transconjugant (data not shown). This primer was therefore replaced by TC1.1R (5'-CGTCTGAATCGTTGATCTATAAG-3') in PCRs on BM4105RF and transconjugant genomic DNA. The 1 Kb Plus DNA Ladder (Invitrogen) was used as marker.

## Abbreviations

MLST: Multi Locus Sequence Typing; CC: Clonal Complex; MSCRAMM: Microbial Surface Components Recognizing Adhesive Matrix Molecules; ST: Sequence Type; MIC: Minimum Inhibitory Concentration; SNP: Single Nucleotide Polymorphism; COG: Cluster of Orthologous Groups; GAP: Glyceraldehyde-3-phosphate; PEP: Phosphoenolpyruvate; CDS: Coding Sequence(s); CRISPR: Clustered Regularly Interspaced Short Palindromic Repeats; IS: Insertion Sequence; PAI: Pathogenicity Island; PFGE: Pulsed-Field Gel Electrophoresis; JCVI: J. Craig Venter Institute; BHI: Brain Heart Infusion; PBS: Phosphate Buffered Saline; BCM: Baylor College of Medicine.

## Authors' contributions

WvS, MJMB, and RJW designed research. JT, JEPV, CMES and APAH performed experiments. WvS, DRR, JB, IJN, and HT analyzed data. WvS, DRR, MJMB, HT, and RJW wrote the paper. All authors read and approved the final manuscript.

## Supplementary Material

Additional file 1 **Supplementary figure - plasmid content of sequenced strains**. The figure shows the presence of large plasmids in the sequenced *E. faecium *strains as determined by S1 nuclease PFGE. Click here for file

Additional file 2 **Supplementary table - overview of COGs present in *E. faecium *genomes**. Table in Excel-format of the COGs present in the seven sequenced *E. faecium *genomes. The first worksheet gives an overview of the number of proteins that were assigned to COGs in each genome sequence. The other worksheets give the proteins (identified by locus tags) that were assigned to COGs for each genome sequence. Click here for file

Additional file 3 **Supplementary figure - graphical overview of COG superfamilies in the *E. faecium *genomes**. The figure shows the classification into COG superfamilies of the proteins encoded by the sequenced genomes. Click here for file

Additional file 4 **Supplementary table - antibiotic resistance genes in the sequenced *E. faecium *isolates**. Table in Excel format of antibiotic resistance phenotypes and the associated antibiotic resistance genes in the sequenced *E. faecium *isolates. Click here for file

Additional file 5 **Supplementary table - COGs present in *E. faecium *and absent from *E. faecalis***. Table in Excel format with COGs that are present in all *E. faecium *genomes sequenced in this study and that are absent from all six available *E. faecalis *genome sequences. Click here for file

Additional file 6 **Supplementary table - COGs present in *E. faecalis *and absent from *E. faecium***. Table in Excel format with COGs that are present in all six available *E. faecalis *genome sequences and that are absent from the *E. faecium *genomes sequenced in this study. Click here for file

Additional file 7 **Supplementary table - the number of shared CDS between the different *E. faecium *isolates**. Table in Excel format with the number of CDS shared between *E. faecium *isolates. Of all possible pairwise comparisons of the seven strains described in this study the total number of shared CDS and the percentage shared CDS relative to the smallest of the two compared genomes is indicated. Click here for file

Additional file 8 **Supplementary table - Proteins unique to infectious *E. faecium *isolates**. Table in Excel format of the proteins that are conserved in the four infectious isolates (E1162, E1636, E1679, and U0317) and which are absent in the non-infectious isolates (E980, E1039, and E1071). Click here for file

Additional file 9 **Supplementary figure - Sequence alignment of the 54 bp repeat flanking genomic islands integrated in the *rpsI *locus**. This figure shows the sequence alignment of the imperfect 54 bp repeat that is flanking genomic islands that have integrated in the *rpsI *locus. Click here for file
