# Supplementary material for: Pyrosequencing-based comparative genome analysis of the nosocomial pathogen Enterococcus faecium and identification of a large transferable pathogenicity island
Source: BMC Genomics. 2010 Apr 14;11:239. doi: 10.1186/1471-2164-11-239 (PMC2858755; doi:10.1186/1471-2164-11-239)
Supplement: Additional file 1 — Supplementary figure - plasmid content of sequenced strains. The figure shows the presence of large plasmids in the sequenced E. faecium strains as determined by S1 nuclease PFGE. [file 1471-2164-11-239-S1.PDF]

Additional file 1: Plasmid profiles of the seven sequenced *E. faecium* strains

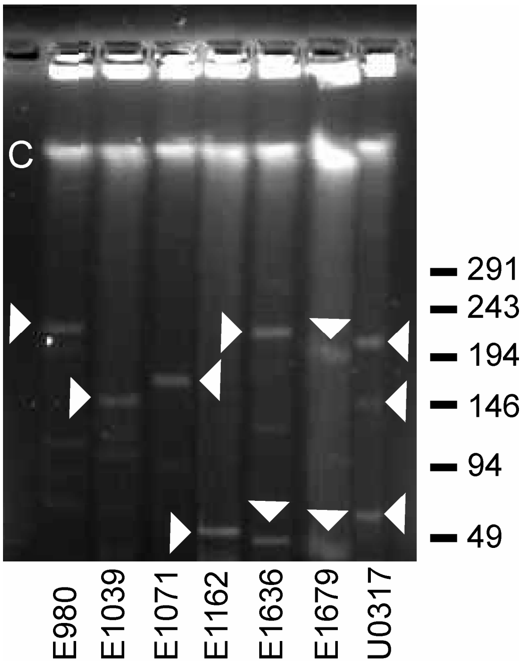

Plasmid profiles (determined by S1 nuclease digestion of total genomic DNA) of the seven *E. faecium* strains of which the genomes were sequenced in this study. Plasmid bands are indicated by the white triangles. Chromosomal DNA is indicated by C.
