# Supplementary material for: Pyrosequencing-based comparative genome analysis of the nosocomial pathogen Enterococcus faecium and identification of a large transferable pathogenicity island
Source: BMC Genomics. 2010 Apr 14;11:239. doi: 10.1186/1471-2164-11-239 (PMC2858755; doi:10.1186/1471-2164-11-239)
Supplement: Additional file 3 — Supplementary figure - graphical overview of COG superfamilies in the E. faecium genomes. The figure shows the classification into COG superfamilies of the proteins encoded by the sequenced genomes. [file 1471-2164-11-239-S3.PDF]

Additional file 3: COG classification of proteins encoded by the sequenced *E. faecium* genomes

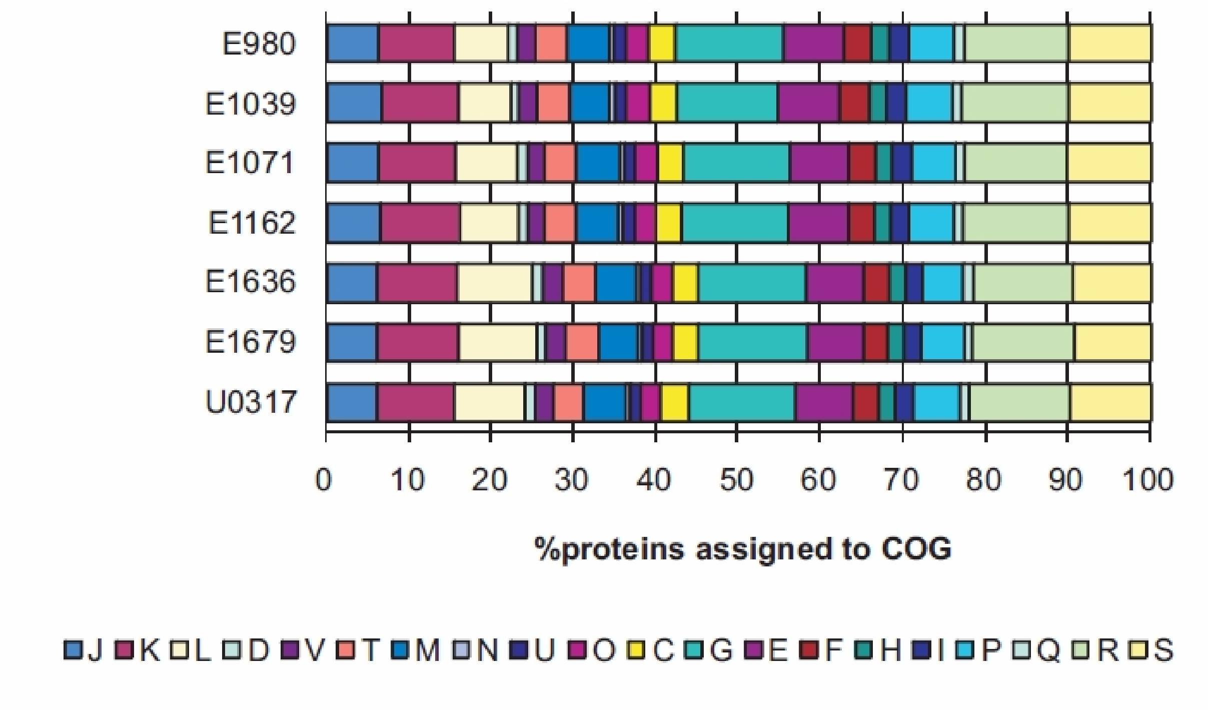

The one-letter codes represent the following COG functional categories: J: translation, ribosomal structure and biogenesis; K: transcription; L: replication, recombination and repair; D: cell cycle control, cell division, chromosome partitioning; V: defense mechanisms; T: signal transduction mechanisms; M: cell wall/membrane/envelope biogenesis; N: cell motility; U: intracellular trafficking, secretion, and vesicular transport; O: posttranslational modification, protein turnover; chaperones; P: inorganic ion transport and metabolism; C: energy production and conversion; G: carbohydrate transport and metabolism; E: amino acid transport and metabolism; F: nucleotide transport and metabolism; H: coenzyme transport and metabolism; I: lipid transport and metabolism; Q: secondary metabolites biosynthesis, transport and catabolism; R: general function prediction only; S: function unknown.
