# Supplementary material for: Pyrosequencing-based comparative genome analysis of the nosocomial pathogen Enterococcus faecium and identification of a large transferable pathogenicity island
Source: BMC Genomics. 2010 Apr 14;11:239. doi: 10.1186/1471-2164-11-239 (PMC2858755; doi:10.1186/1471-2164-11-239)
Supplement: Additional file 9 — Supplementary figure - Sequence alignment of the 54 bp repeat flanking genomic islands integrated in the rpsI locus. This figure shows the sequence alignment of the imperfect 54 bp repeat that is flanking genomic islands that have integrated in the rpsI locus. [file 1471-2164-11-239-S9.PDF]

Additional file 9: Alignment of the imperfect 54-bp repeat at the *rpsI* locus

```
                      ********** ** ** ** ******** ** ** ** ** *********
E1162-rpsI        AAAAAACCAGGTCTCAAAAAGGCTCGTAAAGCATCTCAATTCTCTAAACGTTAA
E1162-3′ PAI      AAAAAACCAGGTCTTAAGAAAGCCCGTAAAGCTTCACAGTTCTCAAAACGTTAA
E1679-rpsI        AAAAAACCAGGTCTTAAAAAAGCTCGTAAAGCTTCACAGTTTTCAAAACGTTAA
E1679-3′ PAI      AAAAAACCAGGTCTTAAGAAAGCCCGTAAAGCTTCACAGTTCTCAAAACGTTAA
U0317-rpsI        AAAAAACCAGGTCTTAAAAAAGCTCGTAAAGCTTCACAATTCTCAAAACGTTAA
U0317-3′ PAI      AAAAAACCAGGTCTTAAGAAAGCCCGTAAAGCTTCACAGTTCTCAAAACGTTAA
E1636-rspI        AAAAAACCAGGTCTTAAGAAAGCACGTAAAGCTTCACAGTTTTCAAAACGTTAA
E1636-3′ GI       GTTTAACCAGGTCTTAAGAAAGCCCGTAAAGCTTCACAGTTCTCAAAACGTTAA
E1071-rpsI        AAAAAACCAGGTCTTAAGAAAGCACGTAAAGCTTCTCAATTTTCAAAACGTTAA
E1071-3′ GI       GGCTAACCAGGTCTTAAGAAAGCCCGTAAAGCTTCACAGTTCTCAAAACGTTAA
E1039-rpsI        AAAAAACCAGGTCTTAAGAAAGCTCGTAAAGCATCACAATTCTCAAAACGTTAA
E980-rpsI         AAAAAACCAGGTCTTAAAAAAGCTCGTAAAGCATCACAATTCTCAAAACGTTAA
```

Abbreviations: *rpsI*: repeat-sequence at 3’ end of the *rpsI* gene; 3’ PAI: repeat-sequence at 3’ end of the *esp* pathogenicity island; GI: repeat-sequence at 3’ end of the inserted genomic island. Asterisks indicate completely conserved residues.
